# A Study on the Safety and Effects of *Amorpha fruticosa* Fruit Extract on Spontaneously Hypertensive Rats with Induced Type 2 Diabetes

**DOI:** 10.3390/cimb44060176

**Published:** 2022-06-02

**Authors:** Rumyana Simeonova, Aleksandar Shkondrov, Ekaterina Kozuharova, Iliana Ionkova, Ilina Krasteva

**Affiliations:** 1 Department of Pharmacology, Pharmacotherapy and Toxicology, Faculty of Pharmacy, Medical University of Sofia, 1000 Sofia, Bulgaria; rsimeonova@pharmfac.mu-sofia.bg; 2 Department of Pharmacognosy, Faculty of Pharmacy, Medical University of Sofia, 1000 Sofia, Bulgaria; shkondrov@pharmfac.mu-sofia.bg (A.S.); ionkova@pharmfac.nat.bg (I.I.)

**Keywords:** *Amorpha fruticosa*, invasive plants management, diabetes, hypertension

## Abstract

Metabolic syndrome is characterized by a variety of diagnostic criteria: obesity, dyslipidemia, type 2 diabetes, and arterial hypertension. They contribute to the elevated risk of cardiovascular morbidity and mortality. The potential for *Amorpha fruticosa* L. (Fabaceae) to improve diabetes and metabolic disease is promising, based on in vitro tests. This is why a further investigation of the species is needed. Additionally, a toxicity review in relation to safety revealed that to date, there are no published data regarding the toxicity of *A. fruticosa* towards humans. This species could provide abundant and cheap resources because it is an aggressive invasive plant that grows almost unrestrictedly. The objective of this study was to evaluate the acute toxicity of a purified extract of *A. fruticosa* (EAF), and to assess its antioxidant, antihypertensive, and antihyperglycemic activity in streptozotocin-induced diabetic spontaneously hypertensive rats (SHRs). The EAF was slightly toxic (LD_50_ = 2121 mg/kg, b.w.) when administered orally, and moderately toxic (LD_50_ = 316 mg/kg, b.w.) at intraperitoneal administration, both in mice. The oral administration of EAF (100 mg/kg) for 35 days to SHRs caused significant decreases in the systolic pressure, blood glucose levels, and MDA quantity. It also increased the hepatic level of the endogenous antioxidant GSH, not only in diabetic SHRs, but also in the control group. An additional potential benefit to human health might be conferred through the environmental management of *A. fruticosa* based on its large-scale use for medicinal purposes, as this aggressive invasive species brings problems to natural habitats in many European countries.

## 1. Introduction

Type 2 diabetes contributes to an elevated risk of cardiovascular morbidity and mortality [1,2]. Synthetic hypotensive and hypoglycemic agents that are capable of reducing blood pressure and blood sugar level possess many worrying side effects. Therefore, finding other antihypertensive and anti-diabetic agents, especially those comprising plant sources, is desired. Plants have been investigated all over the world for their potential to treat hypertension, diabetes, and others oxidative disorders [3,4,5]. Thousands of plant species have been screened for their activity. However, the ultimate objective of their use is that they should interact directly with our body chemistry without side effects [6].

The initial phase of all these studies is based on ethnobotanical research. Traditional knowledge regarding diabetes dates back to the Ancient Egyptians and Greeks [7,8], which is related to a long empirical tradition of using plants to treat the condition in the Old World. Ethnobotany provides a large number of plants from Europe, Turkey, and Iran, and these studies are important sources of guidance in the search for new medicines to address globally relevant diseases such as metabolic syndrome and diabetes [9,10,11,12]. The members of Fabaceae are often listed among plants that are used against diabetes [9,10,11,12,13], and that are supported by clinical data [14]. It has been shown that the antidiabetic activity is due to amorfrutins that were first found in, and consequently named after, *Amorpha fruticosa* L. (Fabaceae) [15,16,17,18]. This plant is native to North America [19]. The Seminoles used it as an application against gastric distress and as a tonic in general, and they also mixed it with other plants to treat chronic sickness, although a particular use for it against the symptoms of diabetes has not been reported by the Native Americans [20]. *A. fruticosa* is a good candidate as a source of abundant and inexpensive resources for bioactive substances for new medicines to address diabetes, since during the last few decades it has behaved as an aggressive and invasive plant species, growing almost unrestrictedly in various parts of the world [20]. The plant contains isoflavonoids, and their derivatives are called rotenoids. The second important group of phenolic compounds are prenylated stilbenoids. Among them are amorfrutins, which are quite diverse. The potential for *A. fruticosa* as a treatment for diabetes and metabolic disease is promising, according to in vitro tests, as the application of amorfrutin resulted in a significant reduction in insulin resistance, similar to the reduction that has been observed with the clinical drug rosiglitazone. Additionally, a toxicity review in relation to the safety of *A. fruticosa* revealed that until now, there have been no published data concerning the toxicity of *A. fruticosa* [20].

The objective of this study was, firstly, to evaluate the acute in vivo toxicity of a purified extract of *A. fruticosa* fruits (EAF) in female mice, and secondly, to assess its antioxidant, antihypertensive, and antihyperglycemic activities in streptozotocin-induced diabetic spontaneously hypertensive rats (SHRs).

## 2. Materials and Methods

### 2.1. Plant Material

Mature fruits were collected in October 2018 from a location in Pasarel village, Sofia district (N 42.535218, E 23.521695), and identified by one of the authors (E.K.).

### 2.2. Phytochemical Analysis

The dried plant material (300 g) was pulverized, sieved (3 mm), and percolated exhaustively with dichloromethane (4.5 L). The defatted plant substance was then further extracted with 80% methanol (12 L); the resulting extract was filtered, evaporated on a rotary evaporator, lyophilized, and named EAF.

The analysis of EAF was performed using HPLC with two analytical parameters—amorfrutins (A and B) and total flavonoids. A Young Lin 9100 HPLC system (Hogyedong, Anyang, Korea) equipped with a YL 9101 vacuum degasser, YL 9110 quaternary pump, YL 9131 column compartment, YL 9160 PDA detector, 7725i manual injector, and Clarity (v 2) software was used. Separations were performed on either the Purospher^®^ STAR column (RP C18ec, 4.6 *×* 125 mm, 5 µm, Merck, Darmstadt, Germany) for amorfrutins, or on Luna^®^ (100 Å, 250 × 4.6 mm, 5 µm, Phenomenex, Torrance, CA, USA) for flavonoid determination. For both analyses, EAF was dissolved in MeOH (1 mg/mL) and filtered through a syringe filter (PVDF, 0.45 µm). The injections were made in triplicate, and the mean values were used for calculation. The quantity of the amorfrutins A and B in EAF was examined using a previously reported method [21] with slight modifications after revalidation, and calculated as the percentage of amorfrutin B. The flavonoid content in EAF was determined and expressed as rutin [22].

### 2.3. Animals

An acute toxicity test was performed in 36 female mice (22.5 g ± 3.6 g) obtained from the National Breeding Centre of the Bulgarian Academy of Sciences (Slivnica, Bulgaria). Pharmacological experiments were performed in male spontaneously hypertensive rats (SHRs) of the inbred strain Okamoto-Aoki (initial body weight 200–250 g), which were obtained from Charles River Laboratories (Sulzfeld, Germany). The animals were housed in Plexiglas cages (2 per cage) at 20 ± 2 °C and a 12/12 h light/dark cycle. During the procedure, the rats were given access to essential food and water. All studies were conducted in accordance with the principles stated in the European Convention for the Protection of Vertebrate Animals used for Experimental and other Scientific Purposes (ETS 123) and proven by the Bulgarian Agency of Food Safety (Permission No 187).

### 2.4. Design of the Experiment

Sixteen male spontaneously hypertensive rats (SHRs) were divided into four groups, each consisting of four animals (n = 4). The SHRs were chosen because in this strain, the chemically induced diabetes produces more profound effects than it does in normotensive rats, and they are considered to be a suitable model for the evaluation and examination of oxidative stress, hypertension, and diabetes [23]. The treatment of the SHRs is presented in Table 1.

The animals were observed daily for behavioral changes and signs of toxicity. On day 36, after 35 days of treatment, the animals were anesthetized with ketamine/xylazine (80 mg/10 kg i.p.) and decapitated. The livers were dissected and prepared for the assessment of MDA and GSH levels.

### 2.5. Acute Toxicity Tests

Acute toxicity tests of EAF were performed on 36 ICR female mice (18 for oral toxicity and 18 for intraperitoneal toxicity) using the simplified method of Lorke [24]. EAF was dissolved in 0.9% NaCl and administered orally (p.o.) or intraperitoneally (i.p.) at various doses. The experiment was conducted in two stages. In the first stage, 18 mice were used, and divided into two groups (for p.o. and i.p. administration) of 9 mice each. Each group was divided into three subgroups of three animals treated with different doses (10, 100, and 1000 mg/kg) of EAF, p.o., or i.p. Immediately after the EAF administration, the animals were observed for signs of toxicity and death, and they were subsequently observed every two hours for the next 24 h. In the second phase, another 18 mice were used, divided into 2 groups (for both routes of administration) of 9 animals each. Each group was again divided into three subgroups for the administration of three higher doses of EAF (1500, 3000, and 5000 mg/kg). The animals were observed for 24 h.

The LD_50_ was calculated using the following equation:

(1) $${\mathrm{LD}}_{50}=\sqrt{{(D}_{0}{\;\times \;D}_{100})}$$

where D_0_ is the highest dose that gave no mortality, and D_100_ is the lowest dose that produced mortality [24].

### 2.6. Blood Pressure Measurement

Systolic blood pressure was measured in the tail using an automated device (a CODA non-invasive blood pressure system, Kent Scientific Corporation, Torrington, CT, USA). Prior to the experiment, the rats were warmed for 10 min at (37 °C) to facilitate blood flow to the tail. The mean value of three blood pressure measurements for each rat was used. The SHRs with the highest blood pressure values were used for the in vivo experiment. 

### 2.7. Induction of Type 2 Diabetes Mellitus in SHRs

Type 2 diabetes was induced via an i.p. administration of streptozotocin (STZ) (45 mg/kg b.w.) dissolved in 0.1 M citrate buffer, pH 4.4, 15 min after the administration of nicotinamide (NA) (110 mg/kg b.w.) to the fasting SHRs. After 48 h, diabetes was confirmed by measuring the blood sugar level using an Accu-Chek glucometer (Roche, Mannheim, Germany) in blood taken from the tail vein. Rats with glucose levels of 12 mmol/L or more were considered as diabetic and were included in the study [25].

### 2.8. Measurement of MDA Levels in Liver Homogenate 

Livers from the experimental animals were homogenized with 0.1 M phosphate buffer and EDTA, pH 7.4. Aliquots of the homogenates were heated for 20 min in a water bath (100 °C) with thiobarbituric acid. The number of reactive species formed from the thiobarbituric acid (TBARS) (expressed as MDA equivalents) was measured at 535 nm. The concentration of MDA was calculated using a molar absorbance coefficient of 1.56 × 10^5^ M^−1^ cm^−1^ and expressed as nmol/g tissue [26].

### 2.9. Measurement of GSH Levels in Liver Homogenate 

GSH was evaluated by measuring non-protein sulfhydryls after trichloroacetic acid (TCA) protein precipitation. Livers were homogenized in 5% TCA (1:4) and centrifuged for 20 min at 4000× *g*. The reaction mixture contained 0.05 mL supernatant, 3 mL 0.05 M phosphate buffer (pH = 8), and 0.02 mL DTNB reagent. The absorbance was determined at a wavelength of 412 nm and the results were expressed as nmol/g tissue [27].

### 2.10. Statistical Analysis

Statistical analysis was performed using the MEDCALC program (v. 10.06.). The results are expressed as a mean value ± SD on four rats in each group. The experimental groups were compared using the Kruskal–Wallis variance analysis test, and a post-hoc analysis using the Mann–Whitney *U* test was performed. Values of *p* ≤ 0.05 were considered to be statistically significant.

## 3. Results

### 3.1. Phytochemical Analysis

The amounts of amorfrutins (A and B) and flavonoids were determined in EAF (the values reported are per dry weight of EAF). Although according to the official standardization documents [28] a minor modification regarding the length of the column used (from 100 mm in the original to 125 mm) does not require a revalidation of the method, the procedure was still performed. The specificity was investigated as a ratio over a blank solution. No peaks with the retention time (t_R_) of amorfrutin B were detected. The repeatability was investigated over standard solutions of amorfrutin B. The SD and RSD (in%) were less than 1%. The limits of detection and of quantitation were 0.001 mg/mL and 0.01 mg/mL, respectively. The linearity was investigated over a concentration range of 0.25 to 1 mg/mL amorfrutin B. This response was proportional in these areas (*r^2^* = 0.99). The retention time of amorfrutin B was 15.80 ± 0.5 min (as a mean of three injections), both in the standard solutions and in the extract. Of the contents of amorfrutins A and B in EAF, 1.9% was expressed as amorfrutin B. The total flavonoid content of EAF, calculated as rutin, was 2.5%.

### 3.2. Acute Oral Toxicity of EAF

The results show that all of the animals survived the first phase of the experiment without any apparent symptoms of toxicity (Table 2). Some mortality was seen in the second phase in this experiment. According to the Hodge and Sterner scale [29], the investigated EAF could be classified as being slightly toxic when it was administered orally to female mice (LD_50_ = 2121 mg/kg b. w.).

The acute toxicity test showed that the EAF, when administered orally, was slightly toxic in mice.

The same procedure was performed for intraperitoneal administration. In the first phase of the experiment, only one mouse, which was administered the highest (1000 mg/kg) dose, died (Table 3), giving 33% mortality. In the second phase of the experiment 100% mortality was observed. After administrating the higher doses of EAF (from 1000 to 5000 mg/kg b.w.), symptoms of toxic damage were observed; these were expressed as breathing difficulty, which was initially rapid and then severely delayed, as well as ataxia, a lack of coordinated movements, and tremor. A lethal outcome occurred after severe tonic–clonic seizures 10 to 15 min after the administration of the substance. Hence, for intra-peritoneal administration in mice, the LD_50_ was calculated to be 316 mg/kg b.w.

### 3.3. Changes in Systolic Blood Pressure (SBP)

**Figure 1:**
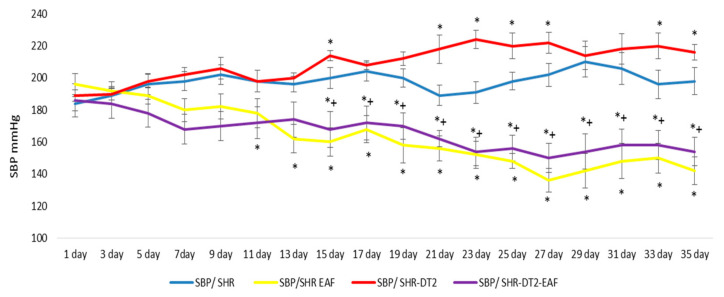
Dynamic changes in systolic blood pressure (SBP). Treatment: as described in the experimental design section. Data are expressed as mean ± SD of four rats (n = 4). For comparison between groups, Mann–Whitney *U* test was performed. * *p* < 0.05 vs. SHR control group; + *p* < 0.05 vs. diabetic SHR group. Abbreviations: SBP, systolic blood pressure; SHRs, spontaneously hypertensive rats; SHR-EAF, SHRs treated with EAF; SHR-DT2, SHRs with streptozotocin-induced type 2 diabetes; SHR-DT2-EAF, diabetic SHRs treated with EAF.

The SBP of the diabetic SHRs was slightly higher throughout the experiment than the control SBP (Figure 1). The treatment of both non-diabetic and diabetic SHRs with EAF decreased the blood pressure in a statistically significant manner (*p* < 0.05), by 32% and 29%, respectively, at the 35th day, compared to the 1st day of the experiment (Figure 1).

### 3.4. In Vivo Changes in Blood Glucose Level

**Figure 2:**
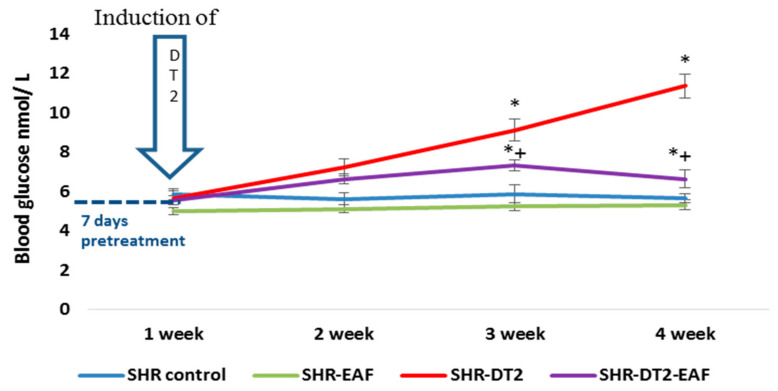
Weekly dynamic changes in blood glucose levels in the experimental groups. Data are expressed as mean ± SD of four rats (n = 4). For comparison between groups, Mann–Whitney U test was performed. * *p* < 0.05 vs. SHR control group; + *p* < 0.05 vs. diabetic SHR group. Abbreviations: SBP, systolic blood pressure; SHRs, spontaneously hypertensive rats; SHR-EAF, SHRs treated with EAF; SHR-DT2, SHRs with streptozotocin-induced type 2 diabetes; SHR-DT2-EAF, diabetic SHRs treated with EAF.

At the end of the experiment, the glucose concentration was significantly elevated in the STZ-induced diabetic SHRs (an 101%, *p* < 0.05), compared to their matched controls (Figure 2). In the diabetic animals, the treatment with EAF resulted in a significant (*p* < 0.05) reduction of 42% for the blood glucose levels (*p* < 0.05), compared to the diabetic SHRs at the 35th day.

### 3.5. Oxidative Stress Markers

**Figure 3:**
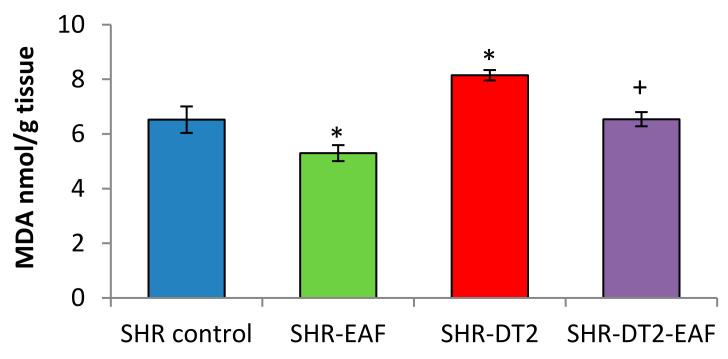
MDA quantity in liver homogenate in all experimental groups. Data are expressed as mean ± SD of four rats (n = 4). For comparison between groups, Mann–Whitney U test was performed. * *p* < 0.05 vs. SHR control group; + *p* < 0.05 vs. diabetic SHR group. Abbreviations: SBP, systolic blood pressure; SHRs, spontaneously hypertensive rats; SHR-EAF, SHRs treated with EAF; SHR-DT2, SHRs with streptozotocin-induced type 2 diabetes; SHR-DT2-EAF, diabetic SHRs treated with EAF.

**Figure 4:**
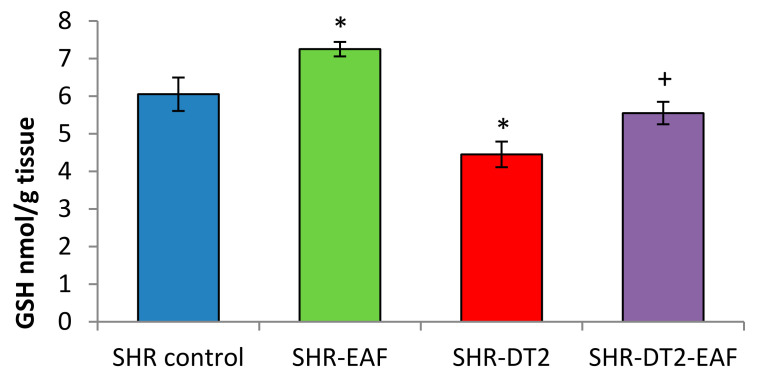
GSH level in liver homogenate in all experimental groups. Data are expressed as mean ± SD of four rats (n = 4). For comparison between groups, Mann–Whitney *U* test was performed. * *p* < 0.05 vs. SHR control group; + *p* < 0.05 vs. diabetic SHR group. Abbreviations: SBP, systolic blood pressure; SHRs, spontaneously hypertensive rats; SHR-EAF, SHRs treated with EAF; SHR-DT2, SHRs with streptozotocin-induced type 2 diabetes; SHR-DT2-EAF, diabetic SHRs treated with EAF.

The levels of the oxidative stress markers are shown in Figure 3 and Figure 4. The administration of EAF leads to a statistically significant decrease of 19% for MDA, and an increase of 20% for GSH in the control SHRs. STZ-induced type 2 diabetes produces an increase of 25% (*p* < 0.05) for MDA, and a decrease of 26% (*p* < 0.05) for GSH. Thirty-five days of oral administration of EAF to the diabetic SHRs restored the levels of oxidative stress markers to near-control values.

## 4. Discussion

There is a great deal of evidence in clinical practice and scientific literature for a direct link between hypertension and diabetes. Common mechanisms, such as the activation of the renin–angiotensin–aldosterone system, oxidative stress, and inflammation, all contribute to the close interrelations of these comorbidities [30].

Systemic hyperglycemia itself leads to an increased rate of formation for reactive oxygen species (ROS), which produces endothelial dysfunction and damage to the micro- and macro-vessels, provoking vasculopathy and aggravating hypertension. The activated renin–angiotensin–aldosterone system in hypertension also induces oxidative stress. Elevated levels of angiotensin II (Ang II) (a pro-inflammatory adipokine) lead to the upregulation of NAD(P)H oxidase through the Ang II type 1 receptor, increase the production of ROS, and are involved, together with endothelial dysfunction, in the pathogenesis of insulin resistance [31,32].

The interaction between hypertension and diabetes is strong, and their combined effects are multiplicative. SHRs are a suitable experimental model for the evaluation and examination of the oxidative stress that underlies the main pathological changes caused by hypertension and diabetes alike [23]. In the field of food science, much interest has been focused on the discovery and development of alternative medicinal foods with natural bioactive compounds that have the ability to decrease oxidative stress in general, as well as high blood pressure and glucose levels.

Among the nutritional factors that improve antioxidant status, and therefore that maintain blood pressure and normoglycemia, the beneficial role of phenolics (including flavonoids, stilbenoides, etc.) has been demonstrated in animals and in humans [33]. Several observations have confirmed the antihypertensive [34], antioxidant, anti-inflammatory, and antidiabetic activities of these compounds [35,36].

Our results demonstrate that the oral administration of EAF for 35 days decreased the blood pressure in control and diabetic SHRs by 32% and 29%, respectively (Figure 1), which is probably due to the presence of antioxidant compounds in the EAF. Different extracts of *A. fruticosa* are rich in phenolic compounds [20], which explains their antioxidant activity. The fruit extract demonstrated in vitro antioxidant inhibition properties [37]. Twelve honey extracts of *A. fruticosa* were investigated, and high levels of DPPH radical scavenging activity, ferric reducing antioxidant power, and ferrous ion-chelating activity were found, which were significantly associated with the total phenolic contents and ascorbic acid contents in this noxious invasive alien plant [38]. The inhibition of oxidative stress could be an additional therapeutic approach to for influencing cardiovascular diseases, including hypertension [31]. In the present work, the antioxidant effect of *A. fruticosa* was confirmed in control and diabetic SHRs, where 35 days of oral administration of EAF decreased the MDA quantity and increased the GSH level. It should be noted that one of the main characteristics of spontaneously hypertensive rats is the increase in oxidative stress described by many research teams [31,32]. For this reason, the extract that we used reduces the content of MDA and increases the level of GHA, both in the control group of hypertensive animals and in the group with induced diabetes. The abatement of oxidative stress, which consumes naturally existing antioxidant components, could reduce blood pressure [39] and hyperglycemia [40]. We speculate that the pronounced antioxidant effect of the species is a key factor for its antihypertensive and antihyperglycemic activity. The PPARγ receptor-binding affinity and the inhibition of the nuclear transcription factor-kB (NF-kB) signaling pathway from amorfrutins, amorphastilbol, etc., which are present in the fruits of *A. fruticosa*, also support our findings, which have revealed that EAF has effective blood glucose-lowering potential in vivo and exerts an anti-diabetic effect on STZ-induced diabetic SHRs.

Until now, the toxic potential of extracts from *A. fruticosa* has only been evaluated using in vitro methods [20]. This was the reason for assessing its toxic potential in experimental animals in order to choose appropriate doses for in vivo investigations.

The lower LD_50_ value in i.p. administration could be attributed to the presence of some cytotoxic rotenoids in EAF [20].

According to the Hodge and Sterner scale [29], the investigated EAF could be classified as being slightly toxic when administered orally to female mice (LD_50_ = 2121 mg/kg, b.w.). The lack of toxicity after the oral administration of the EAF can be explained through two possible scenarios. Probably, during the first passage of the absorbed compounds through the liver, they undergo extensive metabolism and detoxification. The second explanation for the lack of oral toxicity is the lack of enteral absorption of the rotenoids, which are then eliminated from the body through the feces. However, in order to be sure of the reason for the low toxicity, additional studies should be performed on the passage of the biologically active compounds from the EAF through different biological membranes and organs.

## 5. Conclusions

A lyophilized extract from *A. fruticosa* fruits was slightly toxic when administered orally, and moderately toxic after its intraperitoneal administration in mice. EAF demonstrated antioxidant potential, antihypertensive, and antihyperglycemic effects after its oral administration to diabetic and non-diabetic control SHRs.

## Figures and Tables

**Table 1:** SHR groups and their treatments.

SHR Group	Treatment
Group 1 (SHR C)	Treated for 35 days with saline vehicle administered via gavage at 5 mL/kg bw/day; on day 7 of the experiment, the animals received an i.p. injection with citrate buffer (pH 4.5)
Group 2 (SHR-EAF)	treated with EAF (100 mg/kg b.w./day, (1/20 of LD_50_ p.o.) oral-gavage for 35 days
Group 3 (SHR T2D)	Challenged on day 7 of the experiment with nicotinamide (230 mg/kg bw, i.p.) and 15 min after that, with streptozotocin (40 mg/kg b.w., i.p.)
Group 4 (SHR T2D + EAF)	Treated with EAF (100 mg/kg b.w./day, oral-gavage for 7 days); on day 7, the animals were challenged with nicotineamide–streptozotocine (230 mg/kg b.w./40 mg/kg b.w. i.p.) and were treated with EAF for additional 28 days

**Table 2:** Acute oral toxicity of EAF in female ICR mice.

1st Phase		2nd Phase	
Doses mg/kg p.o.	Mortality	Doses mg/kg p.o.	Mortality
10	0/3	1500	0/3
100	0/3	3000	1/3
1000	0/3	5000	3/3

**Table 3:** Acute intraperitoneal toxicity of EAF in female mice.

1st Phase		2nd Phase	
Doses mg/kg i.p.	Mortality	Doses mg/kg i.p.	Mortality
10	0/3	1500	3/3
100	0/3	3000	3/3
1000	1/3	5000	3/3

## Data Availability

Experimental data are available from the corresponding author upon reasonable written request.
